# Epigenetic control of HIV-1 post integration latency: implications for therapy

**DOI:** 10.1186/s13148-015-0137-6

**Published:** 2015-09-24

**Authors:** Amit Kumar, Gilles Darcis, Carine Van Lint, Georges Herbein

**Affiliations:** Department of Virology, Pathogens & Inflammation Laboratory, University of Franche-Comté and COMUE Bourgogne Franche-Comté University, UPRES EA4266, SFR FED 4234, CHRU Besançon, Hôpital Saint-Jacques, 2 place Saint-Jacques, F-25030 Besançon cedex, France; Service of Molecular Virology, Institute of Molecular Biology and Medicine, Université Libre de Bruxelles (ULB), 12 Rue des Profs Jeener et Brachet, 6041 Gosselies, Belgium

**Keywords:** HIV-1, Epigenetics, Latency, Histone modifications, CD4^+^ T cells, Monocyte/macrophage, Microglia, MicroRNAs

## Abstract

With the development of effective combined anti-retroviral therapy (cART), there is significant reduction in deaths associated with human immunodeficiency virus type 1 (HIV-1) infection. However, the complete cure of HIV-1 infection is difficult to achieve without the elimination of latent reservoirs which exist in the infected individuals even under cART regimen. These latent reservoirs established during early infection have long life span, include resting CD4^+^ T cells, macrophages, central nervous system (CNS) resident macrophage/microglia, and gut-associated lymphoid tissue/macrophages, and can actively produce virus upon interruption of the cART. Several epigenetic and non-epigenetic mechanisms have been implicated in the regulation of viral latency. Epigenetic mechanisms such as histone post translational modifications (e.g., acetylation and methylation) and DNA methylation of the proviral DNA and microRNAs are involved in the establishment of HIV-1 latency. The better understanding of epigenetic mechanisms modulating HIV-1 latency could give clues for the complete eradication of these latent reservoirs. Several latency-reversing agents (LRA) have been found effective in reactivating HIV-1 reservoirs in vitro, ex vivo, and in vivo. Some of these agents target epigenetic modifications to elicit viral expression in order to kill latently infected cells through viral cytopathic effect or host immune response. These therapeutic approaches aimed at achieving a sterilizing cure (elimination of HIV-1 from the human body). In the present review, we will discuss our current understanding of HIV-1 epigenomics and how this information can be moved from the laboratory bench to the patient’s bedside.

## Review

Thirty-five million people are living with HIV-1 infection worldwide (UNAIDS, 2014). With the development of effective combined anti-retroviral therapy (cART), mortality and morbidity associated with HIV-1 has been dramatically reduced. cART reduces the plasma viral load below the level of detection of classical assays. However, a persistent residual low-level viremia is observed in most patients using ultrasensitive RT-PCR assays [1, 2]. The quality of life of HIV-1-infected individuals under cART regimen is presumed to be similar to uninfected individuals; however, adverse effect associated with cART is one of the factors responsible for the non-adherence to cART [3]. Even an interruption of cART for a period of few weeks results in a rebound of viremia from the latent reservoirs of HIV-1, and continuous interruption often leads to AIDS. In addition, only a fraction of HIV-1-infected individuals have access to cART making the situation further complicated (WHO) [4]. Several efforts have been made to understand the causes and sources of viral rebound. Outcomes of the studies suggest the involvement of latent reservoirs as a major source of viremia upon cART interruption [2, 5–11].

HIV-1 primarily infects activated CD4^+^ T cells and cells of monocyte/macrophage lineage. HIV-1 infection usually results in the lysis of the CD4^+^ T cells, but on rare occasions, these cells can survive long enough to revert back to a resting memory state [12]. These cells have a long life span and contribute to the persistence of HIV-1 in the infected individuals. On the other hand, macrophages are resistant to cytopathic effect of the virus, and due to their presence in diverse anatomical sanctuaries, they further strengthen the viral persistence. For instance, central nervous system (CNS) is one of the anatomical sanctuaries for HIV-1 latent reservoir. CNS resident macrophages such as meningeal macrophages, perivascular macrophages, macrophages of the choroid-plexus, and microglia are derived and continuously replaced by the migration of monocytes through blood brain barrier [13, 14]. These CNS resident macrophages are susceptible to HIV-1 infection and are largely responsible for HIV-1 associated dementia [15, 16]. The presence of integrated proviral DNA has been also detected in astrocytes [17]. During the late course of HIV-1 infection where CD4^+^ T cells are largely depleted, CNS resident HIV-1-infected cells might represent the source of viral persistence in the infected individuals.

In addition to CNS, the gut plays a pivotal role in the pathogenesis of HIV-1 in patients under cART. HIV-1 transcripts, proviral DNA, and latently infected cells have been isolated from gut-associated lymphoid tissue (GALT) [18–22]. Of note, recently, Rothenberger et al. demonstrated that the viral rebound on treatment interruption is evident at multiple sites with a highly complex and genetically diverse population of virions and suggested GALT as an important latent reservoir [23]. Similarly, the role of gut-associated macrophages in HIV pathogenesis has been also postulated [24]. In the presence of stroma-derived growth factors, HIV-1-infected monocytes can be differentiated into macrophages of lamina propria and could represent a HIV-1 latent reservoir [10, 25].

The mean frequency of latently infected cells in patients on cART, estimated with the viral outgrowth assay, is extremely low (∼1/10^6^ resting CD4^+^ T cells) [26]. These latent reservoirs consist of cells carrying “replication competent, transcriptionally and translationally silenced, extremely stable proviruses” capable of producing virions upon various cellular stimuli [2, 27]. HIV-1 latency has been characterized to large extent in CD4^+^ T cells and to less extent in monocytes/macrophages [2, 10, 11, 28]. The latency is broadly classified into pre and post integration latency [29]. In post integration latency, the integrated proviral DNA is silenced in the target cells by various epigenetic and non epigenetic mechanisms [30, 31]. Epigenetic factors include repressive chromatin structure at HIV-1 promoter by the interplay of several DNA and histone-modifying enzymes. In addition, a growing list of evidences also suggests the relationship between microRNAs and host epigenetic machinery. In the present review, we will limit the discussion to the epigenetic mechanisms responsible for HIV-1 post integration latency and how these mechanisms can be targeted by anti-HIV drugs developed for purging the latent reservoir from HIV-1-infected individuals.

### HIV-1 preferential integration into the host chromatin

Several studies showed the preferential integration of HIV-1 DNA into the euchromatin of the host chromatin in vitro [32, 33] and in vivo [33, 34] suggesting the epigenetic silencing of the provirus. For instance, Han and colleagues explored the integration site in resting CD4^+^ T cells populations isolated from HIV-infected individuals under cART regimen. They investigated 74 integration sites from 16 patients, out of which 93 % resided within transcription units, usually within introns. Integration was random with respect to transcriptional orientation relative to the host gene and with respect to position within the host gene [34]. Similar findings have been also reported in monocyte-derived macrophages (MDMs) [35].

Taken together, these studies suggest the non-random distribution of HIV-1 integration sites in the host genome suggesting the collective efforts of host and viral factors. For example, the lens epithelium-derived growth factor (LEDGF/p75) has been found as an important cellular factor responsible for guiding the pre-integration complex (PIC) to the host chromatin via interaction through integrase (IN) [36–38]. Recently, data from Debyser’s research team suggest the uptake of LEDGF/p75 in the viral particles mediated by IN/pol and specific cleavage by HIV protease [39].The biological relevance of LEDGF/p75 within HIV-1 virion is under investigation [39]. How HIV-1 integration occurs in transcriptional active region has been studied during the last few years. Ocwieja and colleagues observed that the knockdowns of nuclear pore protein RanBP2 and transportin-3 altered integration targeting for HIV in HEK 293 T cells suggesting a link between nuclear pore entry and HIV-1 integration events [40]. A recent study by Marini and coworkers further unfold the mystery of non-random distribution of HIV-1 integration sites in the host chromatin of CD4^+^ T cells [41]. They reported that HIV-1 integration occurs in the outer shell of the nucleus in close correspondence with the nuclear pore. They also showed that functional viral integrase and the presence of the cellular Nup153 and LEDGF/p75 integration cofactors are indispensable for the peripheral integration of the virus [41].

Integration of HIV-1 into the transcriptional units and their subsequent silencing suggests the involvement of transcriptional interference (TI) in the regulation of viral latency. TI refers to the direct inhibitory effect of transcription of one gene on another gene transcriptional process present in cis [42]. The assembly of RNA polymerase complex on integrated HIV-1 5′LTR might be prevented by ongoing transcription of the host gene. The phenomenon of TI has been shown in several cell lines harboring proviral DNA [43–45]. In addition, the role of chromatin reassembly factors (CRFs) has been postulated in regulating the viral gene expression [46].

### HIV-1 promoter: center of HIV-1 epigenomics

HIV-1 viral promoter also called 5′LTR (long terminal repeat) is one of the well-characterized viral elements so far (reviewed in [2]). The 5′LTR has unique blend of robust TATA box, a potent initiator sequence, and binding sites for several transcription factors including nuclear factor kappa B (NF-kB), SP-1, AP-1, LEF-1, COUP-TF, USF, Ets1, and CREB [47–49]. Several studies demonstrated the formation of  heterochromatin structure at 5′LTR of the integrated HIV-1 in different HIV-1 model latent cell lines. Verdin and Van Lint described the nucleosomes organization at 5′LTR in ACH-2 and U1 cell lines under low and high transcription rates. At 5′LTR, there are two precisely positioned nucleosomes named nuc-0 and nuc-1 separated by two nucleosomes free region designated as enhancer/promoter element and a regulatory region termed as HS4 [47]. Nuc-0 is located upstream of the modulator region on HIV-1 whereas nuc-1 is located downstream of the viral promoter and *cis* acting elements [8, 47].

### Histone modifications and HIV-1 post integration latency

Eukaryotic gene expression is largely influenced by chromatin condensation/decondensation [50]. The lightly packed form of chromatin is called euchromatin as opposed to the tightly packed form named heterochromatin. The chromatin condensation status can be modulated through a variety of mechanisms, including post translational covalent modifications of histone tails and recruitment of repressive factors on methylated DNA [2]. These modifications influence gene expression patterns by directly altering chromatin packaging and by generating interactions with chromatin-associated proteins. Of note, integrated HIV-1 is subjected to the same chromatin regulations as for any other cellular genes. An array of DNA and histone modifying enzymes has been described as able to be involved in the latent state of proviral DNA in infected cells.

Histone modifications via methylation and acetylation are well-studied post translational protein modifications involved in regulating HIV-1 latency. These modifications at a particular residue of histone tails can alter accessibility of the transcription factors, viral and RNA polymerizing machinery to the HIV-1 5′ LTR [51]. Histone reversible acetylation is governed by the activity of histone acetyltransferases (HATs) and histone deacetylases (HDACs) (reviewed in [2, 52]). HATs add acetyl group to the ϵ-amino group of lysine residues in histone tails which generally result in active gene expression and compete with HDACs that blunt transcription by reducing accessibility of DNA templates [53, 54]. In cells harboring silenced proviruses, HDACs are recruited to HIV-1 5′ LTR by host factors including late SV40 factor (LSF), ying-yang 1 (YY1), NF-kappaB p50 resulting in hypoacetylation of nuc-1 and configuring the nuc-1 to repressive state [55]. For example, the host factor COUP-TF interacting protein 2 (CTIP2) recruits HDAC1 and HDAC2 to the 5′LTR of viral promoter in monocytes/macrophages [56, 57]. The treatment of latent model cell lines or resting CD4^+^ T cells isolated from HIV-1 infected patients with HDAC inhibitors (HDACi) results in  the induction of HIV-1 transcription [49, 58–61] further strengthening the role of HDAC in the establishment of viral latency.

### Histone and DNA methylation

In addition to histone acetylation-deacetylation, reversible histone methylation is also known to play a role in HIV-1 latency in CD4^+^ T cells and cells of myeloid lineage. Several studies reported silenced proviral DNA with the tri-methylation of histone H3 lysine at position 9 and 27 (H3K9me3, H3K27me3) [56, 57, 62, 63] or dimethylation at lysine 9 (H3K9me2) [64]. These histone modifications result in the condensation of HIV-1 associated nucleosome (nuc1) and thus favor the repression of HIV-1 gene expression. Benkirane and coworkers showed the involvement of Suv39H1 (a histone lysine methyltransferase (HMT)) and HP1 gamma in H3K9me3 and provirus silencing in several cell lines and peripheral blood mononuclear cells (PBMC) isolated from HIV-1-infected patients [62]. Data from Rohr research team further elucidated the involvement of CTIP2 in the recruitment of Suv39H1 to the 5′LTR resulting in H3K9me3 followed by recruitment of HP1-gamma to the viral promoter, formation of heterochromatin, and ultimately HIV-1 silencing in microglial cells [56, 57].

The list of HMTs involved in the regulation of HIV-1 provirus silencing has been growing. For instance, G9a, a HMT, has been shown to promote transcriptional latency of HIV-1 by governing H3K9me2 and formation of repressive chromatin structure at 5′LTR in ACH2 and OM-10.1 cell lines [64]. Similarly, Friedman and colleagues reported the presence of HKMT enhancer of zeste homolog 2 (EZH2) at the silenced 5′LTR in T cell line [65]. EZH2 methyltransferase, a key component of polycomb repressive complex 2 (PRC2), is responsible for the H3K27me3. Interestingly, they observed 5 and 40 % induction in HIV-1 transcription upon SUV39H1 and EZH2 knockdown, respectively, suggesting the prominent role of EZH2 in the regulation of viral latency [65].

DNA methylation is one of the well-studied epigenetic mechanisms in mammals responsible for genomic imprinting, transposon silencing, and differential gene expression [66]. The link between HIV-1 proviral DNA methylation and transcriptional latency is not well characterized and often with contrasting studies. Kauder and colleagues reported the methylation of two CpG islands flanking viral transcription site and association of methyl-CpG binding domain protein 2 (MBD2) and HDAC2 in one of CpG islands in Jurkat cells and HIV-1 latently infected primary CD4^+^ T cells [67]. The role of HIV-1 provirus methylation has been suggested in promoting latency as a late event of latency establishment and could be an additional latency control in addition to histone modifications. Blazkova and colleagues reported high level of DNA methylation in HIV-1 promoter and enhancers in latent reservoir isolated form HIV-1-infected patients with undetectable viremia as compared to viremic patients [68]. Recently, high level of DNA methylation has been reported in PBMCs infected with HIV-1 suggesting DNA methylation as one of the strategies employed by HIV-1 in transcriptional gene silencing [69]. However, several contradictory findings have been also reported. For instance, a study conducted on resting CD4^+^ T cells isolated from aviremic individuals receiving cART revealed rare methylation of HIV-1 proviral DNA [70] suggesting DNA methylation may not be a prominent mechanism responsible for HIV-1 latency. Taken together, further studies deciphering the status of DNA methylation of HIV-1 proviral DNA are required in large cohorts of patients to derive a meaningful conclusion.

### Non-histone epigenetic modifications and HIV-1 post integration latency

Similar to histone proteins, several non-histone proteins which play an important role in HIV-1 transcription are subjected to reversible acetylation and deacetylation [6]. For instance, p300 and CBP acetyltransferase (a member of HATs family) acetylate the NF-kB subunit Rel A/pp65 at lysine residue 218, 221, and 230 [71, 72] and consequently influence not only the DNA binding capacity of this transcription factor but also its ability to interact with IkappaBalpha (IκBα) and ultimately HIV-1 gene expression [6]. The deacetylation of Rel A mediated by HDAC3 and SIRT1 results in the inhibition of HIV-1 gene expression [71, 72]. HDAC3 and SIRT1 deacetylate RelA/pp65 at lysine residue 221 [71, 72] and 310 [73], respectively. The HIV-1 Tat trans-activator is another non-histone protein acetylated by p300, a necessary step in Tat-mediated transactivation, and deacetylated by SIRT1 in vitro and in vivo [74, 75]. The reversible acetylation of Tat is one of the mechanisms of HIV-1 latency regulation [74, 75].

### “Epigenetic drugs” and HIV-1 reactivation as a therapeutic strategy

**Fig. 1:**
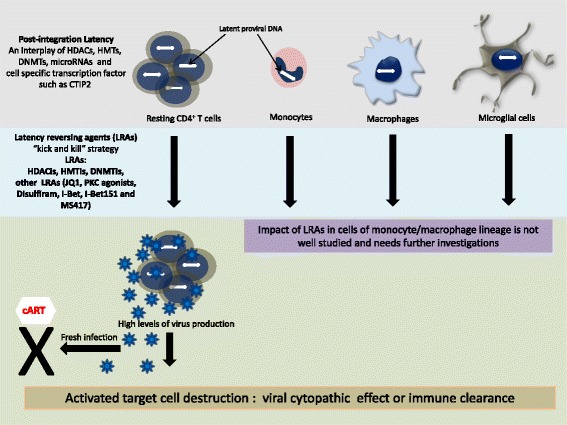
Targeting latent HIV-1 reservoirs. HIV-1 primarily infects CD4^+^ T cells and cells of monocyte/macrophage lineage. Viral latency has been extensively studied in CD4^+^ T cells and to some extent in monocytes/macrophages, microglia , and gut-associated lymphoid tissue macrophages. These latent reservoirs represent the key issue pertaining to the complete eradication of HIV-1 from the infected individuals. According to “kick and kill” strategy, virus can be activated in these reservoirs using a range of latency reversing agents which include HDACis, HMTis, DNMTis, PKC agonists, and several other small molecules. Impact of these LRAs has been well studied in CD4^+^ T cells and to lesser extent in the cells of monocyte/macrophage lineage. Upon reactivation, latent virus undergoes robust replication resulting in production of enormous amount of virus which can induce the lysis of target cells or infected cells can be recognized by the cellular immune clearance machinery. In addition, fresh infection should be stopped by cART. The impact of LRAs in reactivating latent virus in the cells of monocyte/macrophage lineage is not well studied and needs further investigations

Aberrant epigenetic signatures have been reported in several kinds of human pathologies including cancer [76]. The aberrant epigenetic patterns can be corrected by treatments with “epigenetic modifiers” or “epigenetic modifying agents” [77] that are in clinical trials of various phases [76]. The understanding of epigenetic mechanisms associated with the viral latency promotes the development of “epigenetic modifiers” as potential drugs to hopefully eradicate HIV-1 from the infected individuals (sterilizing cure) but more probably to decrease the size of the HIV-1 reservoirs to a level controllable by the host immune system (remission or functional cure). “Kick and Kill” or “shock and kill” [78] is one of the most discussed and tested therapeutic strategy among HIV-1 scientists. “Kick and kill” refers to the induction of latent proviral by various latency reversing agents (LRAs) followed by the elimination of the infected cells by the immune system or by cell lysis induced by viral cytopathic effect and prevention of new infection by cART [5] (Fig. 1). Ideally, LRAs should have permissible toxicity and should not cause a robust and global T cell response [8]. Several “epigenetic modifying agents” including inhibitors of HDAC, HMT, and DNMT have been employed in activating latent reservoir in vitro and ex vivo (Table 1). Recently, pioneering studies have provided evidence that some LRAs can perturb HIV-1 latency in vivo.

### Histone modifiers

#### HDAC inhibitors

HDAC inhibitors (HDACis) are the most studied LRAs. They have been implicated in anticancer research. More than 10 HDACis are tested in various phases of clinical trials for several human pathologies. Vorinostat (SAHA) and valproic acid (VPA) have been approved by food and drug administration (FDA) for treatment of cutaneous T cell lymphoma and neuropsychiatric disorders/epilepsy, respectively [8, 79]. In addition to their low toxic profile, they do not cause a global T cell activation and therefore represent attractive therapeutic molecules [80].

HDAC inhibitors induce HIV-1 expression in latently infected T cells, monocytic cells, and in resting CD4^+^ T cells isolated from HIV-1-infected individuals as reported previously [58, 81–84]. For instance vorinostat has been shown to reactivate HIV-1 in cells derived from infected patients [81, 85, 86]. In addition, in a clinical trial, a single dose of vorinostat has been reported to increase cell-associated unspliced HIV-1 RNA levels within resting CD4^+^ T cells and to increase simultaneously global acetylation [87]. Furthermore, other clinical trials on patients under cART regimen with HDACi vorinostat, panobinostat, and romidepsin have been reported or are ongoing [2, 84, 88, 89] (Table 1). Besides, several other HDACis including sodium butyrate, trichostatin A, oxamflatin, scriptaid, and entinostat have been tested in various cellular models; however, most of them never enter into a clinical trial [84] (Table 1).

**Table 1:** HIV-1 latency reversal agents in various phases of HIV-1 therapeutic development

Latency reversal agent	Class of agent	Agent tested on	Mechanism of action	Stage of therapeutic development	Ref
Vorinostat (SAHA)	HDAC inhibitor	J89 cells and Resting CD4^+^ T cells	Induce acetylation of histone H3K4, H4K4 resulting in remodeling of nuc-1	In vitro, ex vivo and tested in a clinical trial	[81, 87]
Valproic acid	HDAC inhibitor	J-Lat cell lines and U1 cells, patient derived cells	Formation of euchromatin at HIV-1 5′LTR and reactivation of HIV-1 transcription	In vitro, ex vivo, and tested in a clinical trial	[58, 135]
Panobinostat	HDAC inhibitor	CD4^+^ T cells	Formation of euchromatin at HIV-1 5′LTR and reactivation of HIV-1 transcription	Phase 1/2 clinical trial	[136]
Romidepsin	HDAC inhibitor	CD4^+^ T cells	Formation of euchromatin at HIV-1 5′LTR and reactivation of HIV-1 transcription	Ex vivo	[137]
Entinostat	HDAC inhibitor	CD4^+^ T cells, ACH2, and J-lat cell lines	Formation of euchromatin at HIV-1 5′LTR and reactivation of HIV-1 transcription	In vitro, ex vivo	[138, 139]
M344	HDAC inhibitor	J-Lat clones (A7)	Increases histone acetylation and activation of NF-kappaB	In vitro	[140]
Sodium butyrate	HDAC inhibitor	CD4^+^ T cells, J-Lat cell lines, ACH2 and U1 cells	Increases histone acetylation resulting in transcriptional activation of HIV-1 promoter	In vitro	[58, 141]
Trichostatin A	HDAC inhibitor	CD4^+^ T cells, ACH2, and J49 cells	Increases histone acetylation resulting in transcriptional activation of HIV-1 promoter	In vitro, ex vivo	[49, 139]
Oxamflatin	HDAC inhibitor	J89GFP and A7 cell	Increases the acetylation level of histone H3 and histone H4 at the nucleosome 1(nuc-1) site	In vitro	[59, 142]
Scriptaid	HDAC inhibitor	J89GFP and A7 cells	Promotes hyperacetylation of histone	In vitro	[59, 143]
Givinostat (ITF2357 )	HDAC inhibitor	J89GFP, ACH2 and U1 cells	Induces hyperacetylation of histone	In vitro	[59, 144]
CG05/CG06	HDAC inhibitor	ACH2 cells	Induces hyperacetylation of histone	In vitro	[145]
Chaetocin	HMT inhibitor	Resting CD4^+^ T cells isolated from HIV infected patients, ACH-2, OM10.1 cells, infected Jurkat-tat cells	A Suv39H1 inhibitor, induces loss of H3K9me3	In vitro, ex vivo	[64, 92, 93]
BIX-01294	HMT inhibitor	ACH-2 and OM10.1 cells	A G9a inhibitor, promotes repressive H3K9me2	Ex vivo	[64, 93]
3-deazaneplanocin A	HMT inhibitor	Latently infected Jurkat E4 and G4 cells	An inhibitor of EZH2, Induces loss of H3K27me3	In vitro	[65]
5-aza-2′deoxycytidine	DNMTI	ACH-2 cells, U1 cells, and J-Lat cell lines	Inhibits of cytosine methylation and prevent the recruitment of MBD2 and HDAC2 to the 5′LTR	In vitro	[97]
Prostratin	PKC agonist	Patient derived CD4 + T cells, J-Lat cell lines	Activates NF-KB	Ex vivo	[146, 147]
Phorbolmyristate acetate (PMA)	PKC agonist	J-Lat cell lines	Activates NF-KB	Ex vivo	[146, 147]
Diterpene ester ingenol-3-angelate	PKC agonist	U1 cells	Activates NF-KB	In vitro	[148]
Bryostatin-2	PKC agonist	CD4^+^ T-cells, J-Lat cell lines, U1 and OM10.1 cells	Activates NF-KB	In vitro, ex vivo	[149, 150]
JQ1	Unclassified agents	CD4^+^ T cells derived from patient, J-Lat cell lines, U1, ACH2, and OM10.1 cells	Releases BRD4 from the 5′LTR and allows Tat-mediated recruitment of P-TEFb to the 5′LTR.	In vitro and ex vivo	[109, 111]
I-Bet, I-Bet151 and MS417	Unclassified agents	J-Lat cell lines, primary CD4^+^ T cells	Releases BRD4 from the 5′LTR and allows Tat-mediated recruitment of P-TEFb to the 5′LTR.	In vitro	[111]
Disulfiram	Unclassified agents	CD4^+^ T cells	Reactivates latent HIV-1 expression through depletion of the phosphatase and tensin homolog.	Ex vivo, clinical trial	[113–115]

Recent data from Siliciano’s laboratory highlighted the limitation of current latency reversing assay for the evaluation of efficacy of LRAs. They found that except bryostatin-1, none of the LRAs involved in the study (vorinostat, romidepsin, panobinostat, disulfiram, and bryostatin-1) was able to induce the viral outgrowth from the cells isolated form HIV-1-infected aviremic cART-treated patients (Table 1) [90]. There is scarcity of data suggesting the activation of HIV-1 expression upon HDACi treatment in MDMs derived from HIV-1-infected individuals. Interestingly, Jønsson and coworkers reported that romidepsin was able to prevent the de novo infection of PBMCs and CD4^+^ T cells but not in MDMs in vitro [91] and reminded that a complete eradication is only possible by considering all latent reservoirs together.

### Histone methyltransferase inhibitors

Histone methyltransferase inhibitors (HMTs) are other well-studied epigenetic players involved in the maintenance of HIV-1 latency. Various HMTs such as SUV39H1, G9a, and EZH2 are known to play important role in proviral silencing in the latent reservoir. Several studies demonstrated the reversal of HIV-1 latency upon the inhibition of G9a [64, 65, 92, 93], SUV39H1 [92, 93], and EZH2 [65] in model cell lines and cells derived from patients (Table 1). In addition, the treatment of primary resting CD4^+^ T cells with GSK343 (an effective and selective EZH2/EZH1 inhibitor) resulted in reduction of H3K27me3 mark at HIV LTR in the absence of increased proviral expression. Moreover, subsequent treatment of primary resting T cells with HDACi (SAHA or vorinostat) induced a HIV-1 viral production [94]. In combination with HDACi, HMTi could be a therapeutic partner for purging latent HIV-1 reservoirs.

### DNA methyltransferase inhibitors

Several molecules interfering with the DNA methyltransferase activity are in various phases of clinical trials dealing with several kinds of cancers [76, 95, 96]. For instance, Fernandez et al. assessed the combinatorial effect of 5-aza-2′deoxycytidine (Aza-CdR), a DNA methyltransferase inhibitor (DNMTi) and TNF alpha in J-Lat cell lines (6.3, 8.4, 9.2, 10.6), ACH2, J1.1, and U1 cell lines. They observed that among J-Lat cell lines except for J-Lat 10.6, Aza-CdR plus TNF alpha activated HIV at least twice as compared to TNF alpha. On the other hand, in J-Lat10.6 cell line, Aza-CdR plus TNF alpha combination moderately decreases viral activation as compared to TNF alpha alone (Table 1). In contrast, in ACH-2, U1, and J1.1 cells, TNFalpha stimulation with Aza-CdR treatment resulted in a decreased HIV-1 production as compared to the treatment with TNFalpha alone [97]. Similarly, Kauder and colleagues reported the synergism between TNFalpha and Aza-CdR in multiple J-Lat cell lines (J-Lat 6.3, J-Lat 8.4, J-Lat 9.2, and J-Lat 15.4) [67]. Taken together, these results suggest a differential impact of DNMT inhibitors on different HIV-1-infected cellular targets and should be further tested ex vivo in latently infected primary cells derived from patients.

### Other latency reversal agents

HIV-1 latency is a multifactorial phenomenon involving epigenetic machinery and is also subjected to “indirect” epigenetic mechanisms. Besides “direct epigenetic modifiers,” several other molecules have been tested and found effective in recovering HIV-1 from latent reservoirs.

Interleukins (IL) -2 [98] can reverse HIV-1 latency in infected patients but with limited success. On the other hand, IL-7 increases viral production in productively infected cells without disrupting the latency [99]. In a clinical trial, patients receiving IL-2 were found to have low number of latent cells; however, rebound viremia was observed upon cessation of IL-2 treatment [98]. Other potent molecules have been tested for their potential as LRAs. For instance, protein kinase C (PKC) agonists such as phorbol myristate acetate, prostratin, ingenol B are known to induce the HIV-1 expression in latently infected T-cell lines, monocytic cell lines, and patients-derived primary cells [100, 101] (Table 1). Some of these PKC agonists have either potent tumor promoting effect or induce robust global T cells activation [5, 8, 102]. Mechanisms of action of PKC agonists are quite diverse; however, they are known to relocate active NF-kB into the nucleus and to activate the positive transcription elongation factor b (P-TEFb) [8]. In addition, PKC phosphorylates HEXIM1 which may represent one of the key regulatory steps of P-TEFb activity [103]. One important PKC agonist, bryostatin-1, has several advantages over other PKC activators. Bryostatin-1 reactivates latent reservoir without activating T cells, and its pharmacological and toxicological profile are well known [8]. Interestingly, PKC agonists are known to downregulate the expression of CD4 receptor and coreceptor in uninfected cells whereas act as LRA in latent reservoirs. Therefore, treatments with PKC agonists in one hand can reactivate the HIV-1 in infected cells and on the other hand can prevent the new infections [2, 8]. In contrast, Contreras and colleagues reported that the inhibition of PKC delta restricts the replication of R5-tropic viruses in MDMs [104].

Another protein that has gained considerable attention in the last decade is bromodomain-containing protein 4 (BRD4) [105], a double bromodomain that competes with the viral protein Tat for the binding of the P-TEFb complex necessary for the efficient transcription of proviral DNA [105–107]. Indeed, HIV-1 Tat protein recruits P-TEFb complex to the 5′LTR resulting in the phosphorylation of C-terminal domain of RNA polymerase II and efficient transcription elongation [106, 108]. Inhibition of BRD4 by the selective inhibitor JQ1 has been shown to activate HIV-1 transcription in resting CD4^+^ T cells isolated from patients under cART but also in latently infected monocytic and T cell lines [109]. JQ1 releases BRD4 from the 5′LTR and allows the Tat-mediated recruitment of P-TEFb to the 5′LTR [110]. Several other BRD4 inhibitors including I-Bet, I-Bet151, and MS417 have been shown able to reactivate HIV from latency in T cell lines and primary T cells [111]. Moreover, Boehm and colleagues found that knockdown of BRD2, another bromodomain containing protein, resulted in reversal of latency and could be used as a novel therapeutic target [111].

Disulfiram (bis(diethylthiocarbamoyl) disulfide) (DSF), an inhibitor of acetaldehyde dehydrogenase, is a FDA approved compound used to support the treatment of chronic alcoholism by producing an acute sensitivity to ethanol [112]. DSF reactivates HIV-1 in several model latent cell lines and primary CD4^+^ T cells without activating T cells [113, 114] via Akt pathway through depletion of PTEN [114]. In a recent clinical trial, treatment with DSF does not reduce the size of latent reservoir in patients under cART regimen [115]. Of note, one of the limitations of all these so far tested LRAs including “epigenetic modifiers” is their non-specificity towards latently infected cells which may severely affect bystander cells also [8].

### MicroRNAs and HIV-1 latency

MicroRNAs are 20–22 nucleotide long non-coding RNAs encoded by eukaryotic genomes, act as one of the key regulators of post transcriptional gene regulation [116]. In addition, several viral genomes also encode microRNAs and also utilize cellular microRNAs to govern their pathogenesis. Role of microRNAs in contributing to viral latency has been shown in several viruses including human cytomegalovirus [117] and HIV [118]. Bioinformatics analysis of HIV-1 genome predicted the presence of at least 10 microRNAs [118]. Although the presence of microRNAs in HIV-1 is still today a highly controversial issue, however, accumulating evidences suggest the presence and biological relevance of microRNAs in HIV-1 pathogenesis [119–125] and in enforcing latency [123, 124]. For instance, Klase and colleagues demonstrated the presence of viral microRNA derived from the processing of HIV-1 TAR element by Dicer enzyme. Furthermore, they detected the presence of TAR derived microRNA in infected CD4^+^ T cells which could repress the expression of viral genes through transcriptional gene silencing [126]. In another instance, Ouellet and colleagues suggested the biogenesis of two microRNAs (miR-TAR-5 p and miR-TAR-3p)derived from the TAR element upon asymmetrical processing by Dicer in HIV-1 infected cell lines and CD4^+^ T cells infected with HIV-1 [127].

Not only viral microRNAs participate in enforcing latency, but cellular microRNAs also play a pivotal role in governing viral latency. For example, high levels of microRNAs (miR-28, miR-125b, miR-150, miR-223, and miR382) have been reported in resting CD4^+^ T cells as compared to the activated CD4^+^ T cells. The silencing of these microRNAs with anti-sense inhibitors resulted in increased viral production in latent cell lines and resting CD4^+^ T cells [116]. Interestingly, the role of these microRNAs in HIV-1 infectivity of monocytes is also suggested. The lower susceptibility of monocytes and higher susceptibility of macrophages for HIV-1 infection is also linked with the high and low expression of these microRNAs in monocytes and macrophages, respectively [121, 123, 128].

Similarly, the expression of miR-198 is reported to be downregulated during monocytes differentiation into macrophages [129]. MiR-198 does not target the viral transcripts but targets HIV-1 Tat cofactor, cyclin T1, and overexpression of miR-198 in macrophages suppresses HIV-1 replication [129]. The expression of miR-198 is quite low in resting CD4^+^ T cells [130]. In resting CD4^+^T high levels of microRNAs targeting cyclin T1 transcript such as miR-27b, miR-29b, miR-150, and miR-223 have been reported [130]. Another Tat cofactor, P300/CBP-associated factor (PCAF) is targeted by the polycistronic miRNA cluster miR-17/92. In addition, HIV-1 actively suppresses the expression of this microRNA cluster in latent cell lines and PBMCs-isolated form HIV-1-infected patients [131]. In monocytes, high expression of microRNAs (miR-15a, miR-15b, and miR-16) suppresses the expression of purine-rich element binding protein α, another Tat cofactor [132]. The expression of another microRNA miR-29a has been shown to correlate inversely with HIV replication in vitro and ex vivo [133]. More recently, role of microRNA-155 is shown in regulating latency in vitro [134]. Taken together, the emerging data suggest that the microRNAs regulate the HIV-1 latency by directly targeting viral transcripts or by indirectly cellular factors transcripts important for viral replication.

## Conclusions

The interplay of host epigenetic players including HDAC, HMT, and DNMT are largely responsible for the maintenance HIV-1 latency. Latent reservoirs including resting CD4^+^ T cells, monocyte/macrophage lineage, microglia, and gut-associated lymphoid tissue macrophages are the main obstacle in the race for a cure of HIV-1 infection. One of the strategies to eradicate the virus consists of reactivating the latent reservoirs in order to expose the latently infected cells to the immune system and to the viral cytopathic effects while maintaining cART to avoid new infections (“kick and kill strategy”) (Fig. 1). Several efforts have been made in this direction. Various molecules including epigenetic modifying agents such as HDACi, HMTi, and DNMTi have been shown to reactivate the virus in vitro, ex vivo, and in vivo. Indeed, two clinical trials have recently demonstrated that administration to aviremic-treated patients of a single [87] or multiple [89] clinically tolerable dose(s) of vorinostat was associated with an increase expression of cell-associated unspliced HIV-1 RNA levels within resting CD4^+^ T cells in vivo. Importantly, another recent pilot clinical trial showed that panobinostat produced not only an increase of plasma HIV genomic RNA level but also a transient decrease in total HIV DNA level in cART treated patients. In addition to epigenetic modifying agents, several small molecules such as JQ1 and PKC agonists are able to reactivate the latent reservoirs. Each of these agents has their own merits and demerits and not only short- and long-term toxicities but also anti-latency activities on the various viral reservoirs have to be considered in order to develop such LRAs. Indeed, most of the mechanistic and clinically relevant data of HIV-1 epigenomics has been derived from CD4^+^ T cells and to lesser extent from monocytes/macrophages (Fig. 1). Results are encouraging and in future optimized targeting of HIV-1 latent reservoir by several complementary strategies could eradicate HIV-1 infection in patients.

## Abbreviations

AIDS: acquired immune deficiency syndrome
BRD4: bromodomain-containing protein 4
cART: effective combined anti-retroviral therapy
CNS: central nervous system
CRFs: chromatin reassembly factors
CTIP2: COUP-TF interacting protein 2
CTLs: cytotoxic T lymphocytes
DNMT: DNA methyltransferase
DNMTi: DNA methyltransferase inhibitor
DSF: disulfiram
EZH2: enhancer of zeste homolog 2
FDA: food and drug administration
GALT: gut associated lymphoid tissue
HAT: histone acetyltransferase
HDAC: histone deacetylase
HDACi: HDAC inhibitor
HIV-1: human immunodeficiency virus type 1
HMT: histone lysine methyltransferase
HMTi: histone methyltransferase inhibitor
IL: interleukin
IN: integrase
LRA: latency reversing agent
LEDGF/p75: lens epithelium-derived growth factor
LSF: late SV40 factor
LTR: long terminal repeat
MBD2: methyl-CpG binding domain protein 2
MDM: monocyte derived macrophages
NF-kB: nuclear factor kappa B
PBMC: peripheral blood mononuclear cells
PIC: pre-integration complex
PKC: protein kinase C
PRC2: polycomb repressive complex 2
PTEb: positive transcription elongation factor b
TI: transcriptional interference
VPA: valproic acid
WHO: World Health Organization
YY1: ying-yang 1
